# Deciphering sepsis molecular subtypes using large-scale data to identify subtype-specific drug repurposing

**DOI:** 10.64898/2026.03.28.714506

**Published:** 2026-03-30

**Authors:** Leslie A. Smith, Beulah Augustin, Vinitha Jacob, Lauren P. Black, Andrew Bertrand, Charlotte Hopson, Emilio Cagmat, Susmita Datta, Srinivasa T. Reddy, Faheem W. Guirgis, Kiley Graim

## Abstract

Sepsis is a life-threatening dysregulated response to infection, the heterogeneity of which precludes effective targeted therapies. To address this, we created a transcriptomic atlas of publicly available adult sepsis data, on which we performed molecular subtyping and identified potential subtype-specific drug repurposing opportunities. In total, we harmonized data from 3,713 samples across 28 datasets, of which 2,251 were from sepsis patients. Using this data, we identified four molecular subtypes of sepsis (C1 – C4) by clustering the sepsis samples based on expression differences in immune-and lipid-related genes. We next identified gene signatures unique to each molecular subtype. Pathway analysis of these signatures revealed patterns of immune exhaustion and metabolic dysregulation in C1, suggesting potential benefit from corticosteroid treatment. C2 had the youngest patient population and the lowest mortality, and C2 expression patterns were often anti-correlated with those of C1. C3 was enriched for inflammatory and cellular stress pathways, while the highest mortality subtype, C4, showed evidence of immunosuppression and metabolic reprogramming. Gene and pathway-level analysis of our molecular subtypes statistically correlated with results from analysis of 28-day mortality, with the best (C2) and worst subtypes (C4) exhibiting similar molecular dysregulation as survivors and non-survivors, respectively. For each subtype, we then evaluated potential targeted therapies. Using a large-scale pharmacogenomics database, we identified drugs targeting the subtype gene signatures and assessed the potential clinical impacts of these drugs. We identified several potential candidate therapies for each molecular subtype, including possible responsiveness to Methylene Blue therapy for patients from our highest mortality subtype, C4.

Notably, our drug repurposing analysis revealed a significant representation of anti-inflammatory monoclonal antibody therapies across molecular subtypes. The anti-correlated signatures in C1 and C2 suggest that monoclonal antibody therapies may not be effective for patients in both subtypes, which may explain why prior clinical trials have been unsuccessful. Altogether, our detailed molecular subtyping and analysis identify potential drug targets within each molecular subtype, with implications for future precision medicine for sepsis.

## Full Text Availability

The license terms selected by the author(s) for this preprint version do not permit archiving in PMC. The full text is available from the preprint server.

