# Supplementary material for: Deciphering sepsis molecular subtypes using large-scale data to identify subtype-specific drug repurposing
Source: bioRxiv. 2026 Sep 28:2026.03.28.714506. Originally published 2026 Mar 30. Preprint. [Version 2] doi: 10.64898/2026.03.28.714506 (PMC13060274; doi:10.64898/2026.03.28.714506)
Supplement: Supplementary file 1 [file NIHPP2026.03.28.714506v2-supplement-1.pdf]

bioRxiv preprint doi: https://doi.org/10.64898/2026.03.28.714506; this version posted September 28, 2026. The copyright holder for this preprint (which was not certified by peer review) is the author/funder, who has granted bioRxiv a license to display the preprint in perpetuity. It is made available under a CC-BY 4.0 International license.

Complete Supplemental Tables are located in Sepsis_Final

Supplemental Table 1. Harmonized metadata for all samples included in the data atlas.
Harmonized metadata from all datasets included in the data atlas. This harmonized dataset includes all samples (3,713 samples) used for initial batch correction.

Supplemental Table 2. Cluster analysis method comparison.
Results from the clustering runs of the three clustering methods compared in this analysis (K-Means, PAM, Hierarchical Clustering).

Supplemental Table 3. Gene signatures for each molecular subtype.
Differential expression results for the genes assigned to each molecular subtype. Genes assigned to each molecular subtype were similarly differentially expressed in all pairwise comparisons expression comparisons (see Methods).

Supplemental Table 4. Molecular subtype pathway enrichment.
Pathway enrichment results for each molecular subtype. Pathway analysis was done using Biological Process terms in HumanBase using the global network.

Supplemental Table 5. Statistical Analysis.
All results from statistical analyses done on identified molecular subtypes. This includes contingency table analysis of available demographic data as well as comparisons to previously identified subtypes. For each test done on a subset of samples with available data, a null distribution is provided.

Supplemental Table 6. Molecular subtype-specific drug-gene relationships.
The top 50 most differentially expressed genes in each molecular subtype were queried in ClinPGx to identify possible molecular subtype-specific drug associations. A smaller version of this table is provided in the main manuscript (Table 4), this provides all available information from the drug queries.

Supplemental Table 7. Included dataset summaries and links.
List of all studies included in the data atlas with brief summaries of the study, sequencing type, date published, and paper links.

Figure Legends

Figure 1. Study Pipeline Overview.
We processed 28 studies from 4 sources. After preprocessing each dataset, all datasets are combined, and all samples are included for batch correction. Following batch correction, samples classified as having sepsis and septic shock are clustered and analyzed.

Figure 2. t-SNE and Heatmap of C1-C4 Identified Molecular Subtypes.
(A) t-SNE plot depicting the four molecular subtypes identified by K-Means clustering. (B) Heatmap showing the gene expression of the top 25 genes with the largest log fold change for each molecular subtype. Rows depict gene values and are sorted by molecular subtype, columns are samples and are sorted first by molecular subtype, then by disease (sepsis vs septic shock), then by age, and finally by gender. The heatmap reveals distinct gene upregulation across molecular subtypes.

Figure 3. Molecular subtype Clinical Enrichment.

bioRxiv preprint doi: https://doi.org/10.64898/2026.03.28.714506; this version posted September 28, 2026. The copyright holder for this preprint (which was not certified by peer review) is the author/funder, who has granted bioRxiv a license to display the preprint in perpetuity. It is made available under a CC-BY 4.0 International license.

(A) Violin plots with box plots showing age distribution of each molecular subtype. The dotted horizontal line marks the median age across all molecular subtypes. (B-F) Barplots for clinical variables used in analysis showing distribution of each variable across molecular subtypes. (G) TumorMap showing samples colored by previously defined molecular subtype (if applicable). A TumorMap visualizes pairwise sample-sample molecular similarity within a group of samples across a hexagonal grid in a force-directed and non-overlapping manner and provides a unique overview of disease structure that has not been previously applied to sepsis.

Figure 4. Analysis of Mortality Data.

(A) TumorMap of all samples colored by mortality in patients with available mortality data (n = 1,710). The TumorMap is the same as depicted in Figure 3G but colored by survival. (B) Kaplan Meier survival plot showing survival probability by molecular subtype for patients with available time to mortality data (n = 560). RMST p-values show difference between molecular subtype time to survival VS all other molecular subtype time to survivals. (C) Volcano plot of differentially expressed genes between survival and mortality for patients with available mortality data (n = 1,710).

Figure 5. Pathway enrichment and drug associations for sepsis molecular subtypes.

(A) TumorMap colored by our four defined molecular subtypes with associated up-regulated and down-regulated pathways for each molecular subtype. The TumorMap is the same as depicted in Figure 3G but colored by our defined molecular subtypes. For each molecular subtype's pathways, lighter colors indicate upregulation and darker colors indicate down regulation. (B) Barplots for the top 50 most differentially expressed genes for each molecular subtype in comparison to the average expression of the other three molecular subtype.

Supplemental Figure Legends

Supplemental Figure 1: Cumulative dataset variance.

The relative percent of variance in the dataset captured within a subset of genes. A horizontal line shows where 90% of variance is captured and a vertical line shows 5,000 gene cut off used in the manuscript. Using 5,000 genes captures more than 90% of the total variability in the transcriptomic data.

Supplemental Figure 2: Molecular subtype enrichment compared to previous studies.

Barplots showing the distribution of the molecular subtypes defined in this paper compared to subtypes from previous studies. Previous studies are listed on the x-axis and barplots are color-coded by our molecular subtypes.

Supplemental Figure 3: Samples before and after batch correction.

(A) Samples from all datasets included in analysis in PCA space prior to performing batch correction. (B) Samples from all datasets included in analysis in PCA space after performing batch correction.

Figures

bioRxiv preprint doi: https://doi.org/10.64898/2026.03.28.714506; this version posted September 28, 2026. The copyright holder for this preprint (which was not certified by peer review) is the author/funder, who has granted bioRxiv a license to display the preprint in perpetuity. It is made available under a CC-BY 4.0 International license.

Supplemental Figures

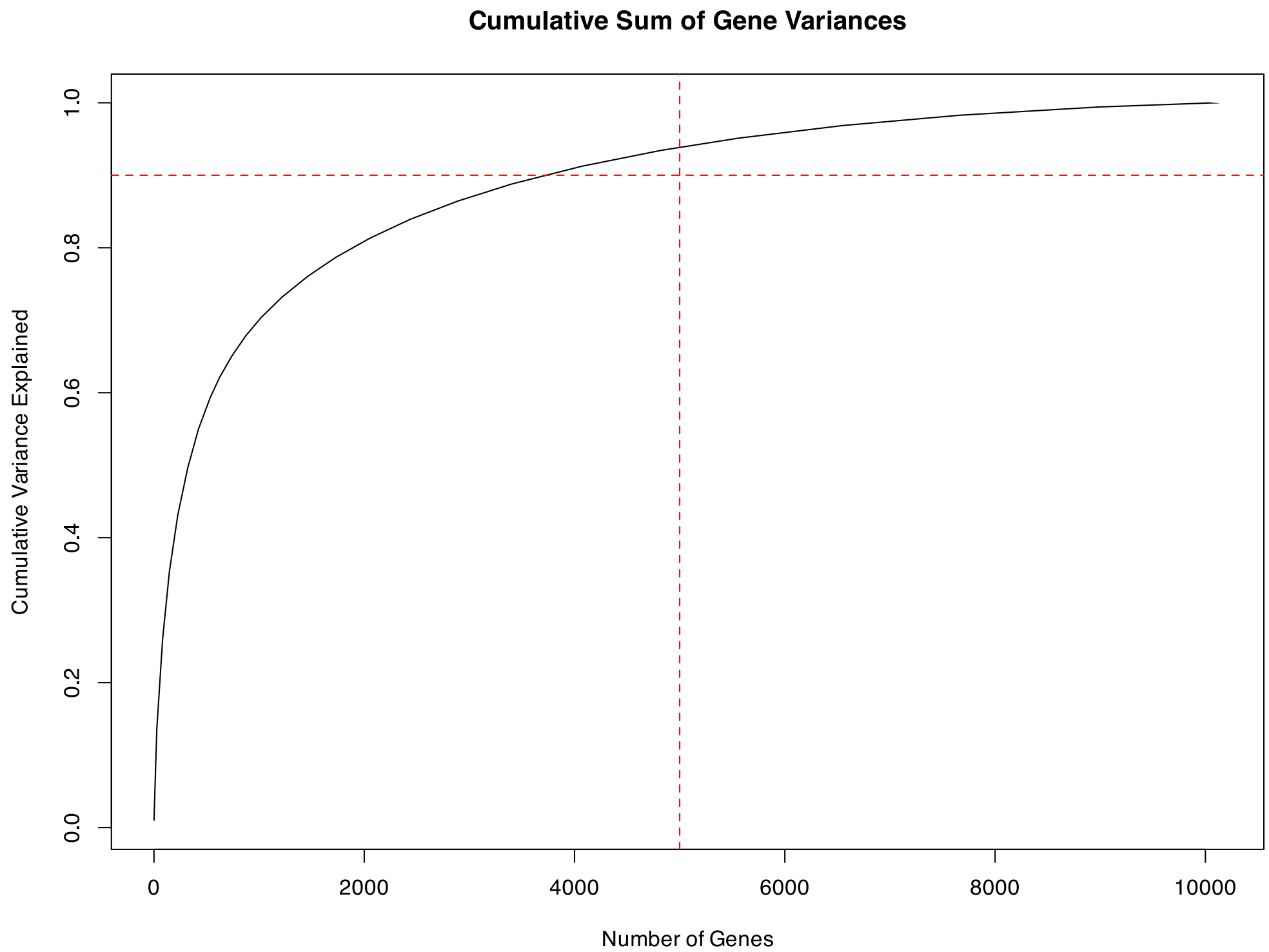

Supplemental Figure 1: Cumulative dataset variance.

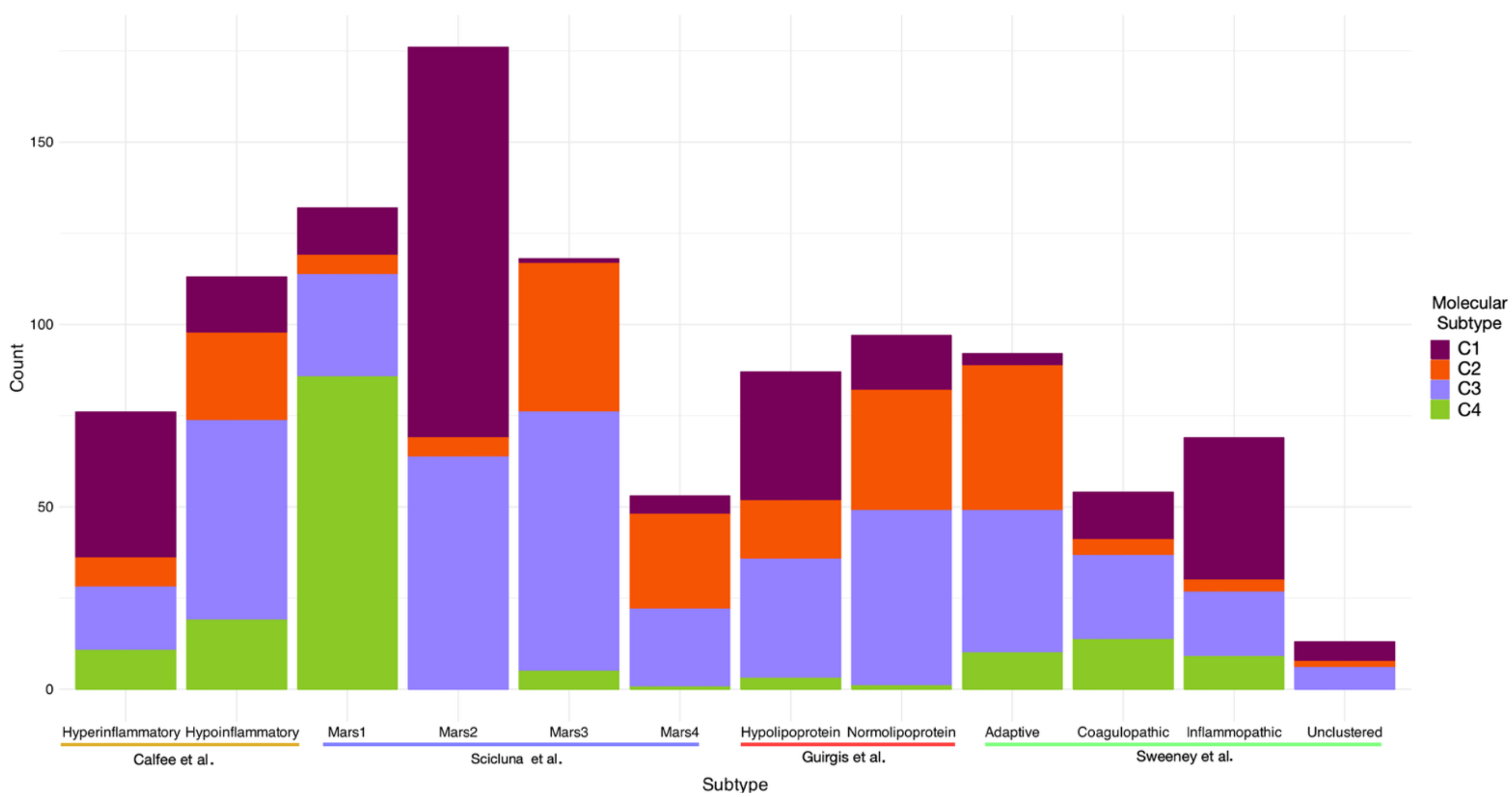

Supplemental Figure 2: Molecular subtype enrichment compared to previous studies.

bioRxiv preprint doi: https://doi.org/10.64898/2026.03.28.714506; this version posted September 28, 2026. The copyright holder for this preprint (which was not certified by peer review) is the author/funder, who has granted bioRxiv a license to display the preprint in perpetuity. It is made available under a CC-BY 4.0 International license.

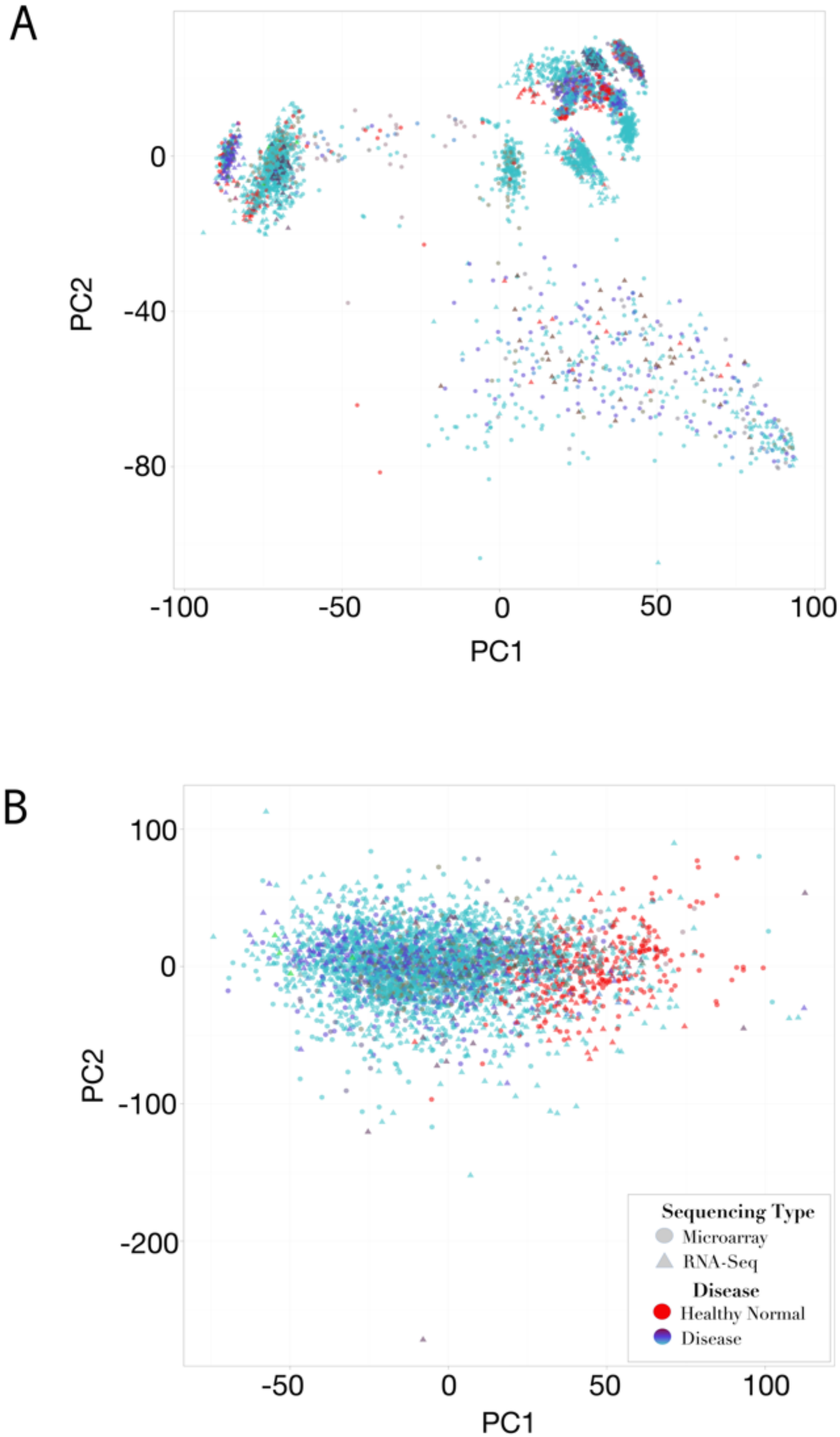

Supplemental Figure 3: Samples before and after batch correction.
